# Myocardial infarction, ST-elevation and non-ST-elevation myocardial infarction and modelled daily pollution concentrations: a case-crossover analysis of MINAP data

**DOI:** 10.1136/openhrt-2016-000429

**Published:** 2016-09-01

**Authors:** Barbara K Butland, Richard W Atkinson, Ai Milojevic, Mathew R Heal, Ruth M Doherty, Ben G Armstrong, Ian A MacKenzie, Massimo Vieno, Chun Lin, Paul Wilkinson

**Affiliations:** 1 Population Health Research Institute and MRC-PHE Centre for Environment and Health, St George's, University of London, London, UK; 2 Department of Social and Environmental Health Research, London School of Hygiene and Tropical Medicine, London, UK; 3 School of Chemistry, University of Edinburgh, Edinburgh, UK; 4 School of GeoSciences, University of Edinburgh, Edinburgh, UK; 5 NERC, Centre for Ecology & Hydrology, Penicuik, UK

**Keywords:** Air pollution, NO<sub>2</sub>, PM

## Abstract

**Objectives:**

To investigate associations between daily concentrations of air pollution and myocardial infarction (MI), ST-elevation MI (STEMI) and non-ST-elevation MI (NSTEMI).

**Methods:**

Modelled daily ground-level gaseous, total and speciated particulate pollutant concentrations and ground-level daily mean temperature, all at 5 km×5 km horizontal resolution, were linked to 202 550 STEMI and 322 198 NSTEMI events recorded on the England and Wales Myocardial Ischaemia National Audit Project (MINAP) database. The study period was 2003–2010. A case-crossover design was used, stratified by year, month and day of the week. Data were analysed using conditional logistic regression, with pollutants modelled as unconstrained distributed lags 0–2 days. Results are presented as percentage change in risk per 10 µg/m^3^ increase in the pollutant relevant metric, having adjusted for daily mean temperature, public holidays, weekly influenza consultation rates and a sine-cosine annual cycle.

**Results:**

There was no evidence of an association between MI or STEMI and any of O_3_, NO_2_, PM_2.5_, PM_10_ or selected PM_2.5_ components (sulfate and elemental carbon). For NSTEMI, there was a positive association with daily maximum 1-hour NO_2_ (0.27% (95% CI 0.01% to 0.54%)), which persisted following adjustment for O_3_ and adjustment for PM_2.5_. The association appeared to be confined to the midland and southern regions of England and Wales.

**Conclusions:**

The study found no evidence of an association between the modelled pollutants (including components) investigated and STEMI but did find some evidence of a positive association between NO_2_ and NSTEMI. Confirmation of this association in other studies is required.

**Key questions**

**What is already known about this subject?**

- Evidence from epidemiological studies examining associations between short-term variations in concentrations of ambient air pollution and acute myocardial infarction (MI) is mixed.
- Most studies have focused on urban populations—few have explored the wider geographical coverage (especially for exposure to PM_2.5_ and its components) offered by the use of atmospheric chemistry transport models (ACTM).

**What does this study add?**

- Using ACTM data, this study found no evidence of an association between the pollutants investigated (O_3_, NO_2_, PM_2.5_, PM_10_ and the PM_2.5_ components sulfate and elemental carbon) and MI or ST-elevation MI.
- However, there was evidence of a positive association between NO_2_ (a traffic related pollutant) and non-ST-elevation MI which appeared to be independent of PM_2.5_ and O_3_.

**How might this impact on clinical practice?**

- Our findings add to the growing epidemiological literature investigating the acute effects of air pollution on cardiovascular health.
- The identification of susceptible population subgroups most at risk on high pollution days would enable appropriate advice to be formulated and communicated in a timely manner to reduce the risk to health.

## Introduction

Air pollution has been associated with adverse cardiovascular heath events in long-term exposure (cohort) studies and in short-term (time-series or case-crossover) studies, and there is mounting evidence from clinical investigations as to potential mechanisms.^1–4^

In terms of the more specific outcome of acute myocardial infarction (MI), epidemiological studies examining the short-term effects of outdoor ambient air pollution have varied in their findings.^5–10^ While a minority of studies suggest an increase in the risk of an MI following exposure to higher concentrations of ozone (O_3_), evidence from the literature of an increase in risk following a rise in exposure to the mass of particulates with an aerodynamic diameter <10 µm (PM_10_), or <2.5 µm (PM_2.5_), nitrogen dioxide (NO_2_), carbon monoxide (CO) and sulfur dioxide (SO_2_) is more consistent.^5–10^ Part of the reason for inconsistencies between studies may be due to the different outcome measures used (from emergency department visits and hospital admissions to mortality), the accuracy of diagnosis or different assumptions about the nature of any association (linear or non-linear in the log relative risk). Results may also be influenced by the relative proportion of MI subtypes in the study population, as well as the source of gasses and the type and source of particle species. The few studies that have presented results for ST-elevation MI (STEMI) and non-ST-elevation MI (NSTEMI) separately have produced conflicting results.^11–13^ An analysis of data (based on 452 343 MI events between 2003 and 2009) from the England and Wales Myocardial Ischaemia National Audit Project (MINAP)^14^ reported small positive associations of NO_2_ (unconstrained distributed lag model (UDLM) lags 0–4) with all MI and NSTEMI,^11^ while a much smaller study in the USA reported a positive association of PM_2.5_ (lag 1 hour) with STEMI.^12^

Previous studies have also varied in the accuracy, completeness and representativeness of the pollution data, the choice of pollution metric and exposure period/lag, as well as to the choice and modelling of potential covariates. Most short-term studies to date, including those based on MINAP data, have used pollution monitoring networks to provide their daily exposure assessments. Few have so far explored the potential advantages of using modelled data that have complete coverage over urban and rural areas, diversity (in terms of particle species components) and enable air quality-related policy scenarios to be investigated. The EMEP4UK atmospheric chemistry transport model (ACTM)^15^ ^16^ is a high-resolution regional application of the well-established European Monitoring and Evaluation Programme (EMEP) MSC-W model.^17^ It simulates the evolution of ambient pollution concentrations using official pollutant emission inventories, relevant natural emissions and driving meteorology, through a detailed treatment of atmospheric chemistry and physics. In this paper, we use ACTM data at 5 km by 5 km spatial resolution to explore associations between modelled ground-level gaseous, total and speciated particulate pollutant concentrations linked to MI, STEMI and NSTEMI events recorded on the England and Wales MINAP database between 2003 and 2010.

## Methods

### Outcome data

The outcome data for the study period of 2003–2010 come from MINAP. This is a register of hospital admissions for acute coronary syndromes (ACS) covering all acute National Health Service hospitals in England and Wales. In addition to discharge diagnosis, the register contains over 100 separate fields of patient-level data, including demographic information (eg, sex, age, smoking status, ethnicity), medical history (eg, history of MI, cerebrovascular disease, peripheral vascular disease, COPD), drug treatment prior to and during the admission and clinical findings (eg, ECG results, symptoms).^14^

For the purpose of this study, we excluded patients with missing geocodes, insufficient information on date of event, missing information on discharge diagnosis or linked to areas outside England and Wales. This left 630 116 events occurring during 2003–2010 and recorded on the MINAP database, of which 203 804 had a discharge diagnosis of STEMI and 323 999 had a discharge diagnosis of NSTEMI (including troponin positive ACS). New left bundle branch block was treated as synonymous with ST-elevation in the diagnosis of STEMI.^18^

### Model pollution and weather data

The pollution data are surface daily outputs (derived from hourly outputs) at 5 km×5 km spatial resolution from an ACTM: EMEP4UK version rv4.3.^15^ ^16^ High resolution is achieved through a nested approach whereby a 5 km×5 km (inner) domain over the British Isles is nested within, and takes boundary and initial conditions from, a larger 50 km×50 km (outer) domain over Europe. The meteorology driving the ACTM comes from the Weather Research and Forecasting (WRF) model version 3.1.1^19^ which is constrained to contemporary meteorological observations, ensuring that the applied meteorology is representative of the real weather conditions prevailing throughout the simulated period. Pollutant and pollutant–precursor emissions over the UK were taken from the UK National Atmospheric Emissions Inventory^20^ and for the outer domain from EMEP estimates provided by the Centre for Emission Inventories and Projections.^21^

The pollutant metrics investigated in this study were daily means of PM_2.5_, PM_10_, sulfate (SO_4_^2−^) and elemental carbon (EC) and daily maximum 8-hour-running mean for O_3_ and daily maximum 1-hour mean for NO_2_. The weather metric was daily mean temperature (see below).

### Monitor pollution data

Monitor data were used only in the assessment of model performance (see below and online supplementary table S1). For this purpose, daily maximum 8-hour running mean O_3_, daily maximum 1-hour NO_2,_ daily mean PM_2.5_ and daily mean PM_10_ were calculated for 2001–2010 using a 75% data capture threshold on hourly data from urban background and rural monitoring sites of the Automatic Urban and Rural Network (AURN) of the UK Department for Environment, Food and Rural Affairs.^22^ PM_2.5_ data were not available for the full period of interest, as the monitoring of this pollutant began only in the latter part of the decade. Between 2001 and 2010 there were changes in the instrumentation used to monitor PM_10_.

10.1136/openhrt-2016-000429.supp1 Supplementary data

### Validation of the pollution and weather models

The EMEP4UK model has undergone extensive validation,^15^ ^16^ ^23^ and their potential for use in epidemiological analyses is beginning to be explored.^24^ For 2001–2010 the average Pearson correlation over time 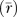 between urban background monitored pollution concentrations at AURN monitoring sites,^22^ and their equivalents for the EMEP4UK model grid incorporating the monitor, was relatively high for daily maximum 8-hour O_3_ (no. of sites (n)=63; 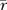
=0.76; SD (r)=0.04) and daily mean PM_2.5_ (n=39; 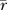
=0.69; SD (r)=0.09) and lower for daily mean PM_10_ (n=57; 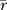
=0.50; SD (r)=0.07) and maximum 1-hour NO_2_ (n=75; 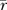
=0.54; SD (r)=0.10).

Imprecision in the estimation of daily pollution concentrations whether modelled or measured may on average lead to some attenuation (ie, bias towards the null) in estimates of the log relative risk obtained from epidemiological analyses. Simple predictions as to the level of attenuation (ie, percentage bias towards the null) that might be expected due to the use of ACTM data are explored in online supplementary table S1.

Since monitored temperature data are available for only a small subset of the model grid boxes, we used the WRF model 2-metre temperature for covariate adjustment. As discussed above, the WRF model was nudged with reanalysis data every 6 hours to closely represent observations such as the surface temperature.

For daily mean temperature, the average model-monitor correlation over time was very high (n=93; 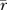
=0.98; SD (r)=0.01) and plots of the relationship between MI and modelled temperature (see online supplementary figure S1) are similar to those previously published by Bhaskaran *et al*,^25^ using the MINAP database and monitored temperature.

### Data linkage

Each MI event was linked to the modelled weather and pollutant exposure data in the 5 km grid closest to the output area (OA) of the patient's residence (using OA centroids rounded to 1000 m to avoid personal identification).

### Statistical methods

The analysis was conducted at the level of the individual using a time-stratified case-crossover analysis.^26^ For each case we defined the index day as the day of the MI and the referent or control days as those days within the same month and on the same day of the week as the event day.^26^ Within each individual we then compared the modelled pollutant exposures between the index and referent days as in a 1:M matched case–control study. The analysis was conducted in STATA V.12 (StataCorp: Stata Statistical Software: Release 12. College Station, TX: StataCorp LP; 2011) using conditional logistic regression. In this way each subject acted as their own control, automatically adjusting for potential non-time varying/time-insensitive confounders such as sex, age, smoking status, socioeconomic status etc. The matched sets or strata were defined in time (ie, year, month, day of the week) to remove trend and seasonal pattern and additional covariates were added to the regression models to adjust for temperature, public holidays, influenza epidemics and residual seasonality. The primary regression model included: two natural cubic splines (each with 5 degrees of freedom (df)) representing mean daily temperature averaged across the day and the day before (mean lag 0–1) and mean daily temperature averaged across days 2–6 before (mean lag 2–6); a binary indicator variable for public holidays; the Royal College of General Practitioners (RCGP) England and Wales weekly consultation rate for influenza-like illness for the week of the event;^27^ and sine and cosine terms representing a simple annual cycle. A priori, pollutants were included in analyses as unconstrained distributed lags 0–2 days (UDLM 0–2). Under the rare disease assumption, ORs from conditional logistic regression were interpreted as relative risks and are presented as such in tables and plots with their 95% CIs.

Possible effect modification by season (autumn=September–November, winter=December–February, spring=March–May; and summer=June–August) and, where applicable, by sex and age group (≤64, 65–74, 75–84, ≥85) was investigated by including appropriate interaction terms in the regression models and testing for an improvement in fit using likelihood ratio tests. However, when investigating effect modification by Government Office Region (10 in England and Wales), a two-stage analysis was employed whereby ORs from region-specific conditional logistic regressions were meta-analysed using METAN in STATA V.12, (StataCorp LP; 2011) to obtain overall relative risks (across all regions), subtotal relative risks for each of three broader areas or ‘super regions’ (ie, the North, Midlands and the South) and tests of heterogeneity between regions and between ‘super regions’. This approach facilitated region-specific covariate adjustment.

Underlying our analyses is the assumption that any association between MI and pollution is approximately log-linear. This assumption was investigated in sensitivity analysis by fitting natural cubic splines (each with 2 df) to simple pollutant averages (averaged over lags 0–2) and testing for non-linearity using the Wald χ^2^ tests (df=1).

## Results

Having excluded records with missing data on exposures or covariates, our main analysis was based on 626 239 events occurring during the study period (ie, 2003–2010) and recorded on the MINAP database. The number of control days ranged from 1 to 4 per case but with <1% of cases matched to only 1 or 2 controls. Of these 626 239 events, 202 550 were diagnosed on discharge as STEMI, 322 198 as NSTEMI and 37 579 as ACS (troponin negative). The remaining 63 912 had discharge diagnoses, including threatened MI, chest pain of uncertain cause, unconfirmed MI, Takotsubo cardiomyopathy, ACS (troponin unspecified), PCI-related MI and other (ie, admitted with clinical suspicion of cardiac pain but diagnosis other than cardiac ischaemia confirmed). Our definition of ALL MI includes all 626 239 events and is therefore broad and inclusive. The median (IQR) age, in years, of cases was 70.6 (59.1–80.0) for ALL MI, 66.2 (56.0–76.5) for STEMI and 74.2 (63.2–82.4) for NSTEMI and the percentage (n/N) male was 65% (404 374/624 926), 70% (141 971/202 024) and 62% (199 216/321 633), respectively.

### Association with pollution

In our primary logistic regression models adjusted for temperature lags 0–1 and 2–6 days, influenza, public holidays and residual seasonality (table 1), there was no evidence of an association of ALL MI or STEMI with any of O_3_, NO_2_, PM_10_ or PM_2.5_. For NSTEMI, there was a statistically significant (p=0.043) positive association with NO_2_ suggestive of a 0.27% (95% CI 0.01% to 0.54%) increase in risk per 10 µg/m^3^ increase in the daily 1-hour maximum concentration. This association did not differ significantly by season (p=0.571), age-group (p=0.986) or sex (p=0.859). It also persisted following adjustment for O_3_, and increased in magnitude following adjustment for PM_2.5_ (p=0.008) (table 1: two pollutant models).

**Table 1:** Estimates for the percentage change in risk (95% CI) per 10 µg/m^3^ increase in pollutant: single and two pollutant models*

Pollutant†	All MI Percentage change (95% CI)	STEMI Percentage change (95% CI)	NSTEMI Percentage change (95% CI)
Single pollutant regression model			
O_3_	−0.06 (−0.29 to 0.17)	−0.16 (−0.57 to 0.25)	−0.05 (−0.37 to 0.28)
NO_2_	0.09 (−0.10 to 0.28)	−0.16 (−0.49 to 0.18)	0.27 (0.01 to 0.54)
PM_2.5_	−0.04 (−0.45 to 0.38)	−0.34 (−1.06 to 0.39)	−0.15 (−0.72 to 0.43)
PM_10_	−0.20 (−0.53 to 0.13)	−0.37 (−0.95 to 0.21)	−0.34 (−0.80 to 0.12)
Two pollutant regression model			
NO_2_ (adjusted for O_3_)	0.07 (−0.13 to 0.27)	−0.23 (−0.57 to 0.12)	0.28 (0.00 to 0.57)
NO_2_ (adjusted for PM_2.5_)	0.14 (−0.08 to 0.37)	−0.07 (−0.46 to 0.32)	0.43 (0.11 to 0.74)
PM_2.5_ (adjusted for O_3_)	−0.04 (−0.46 to 0.37)	−0.36 (−1.09 to 0.37)	−0.15 (−0.73 to 0.43)
PM_2.5_ (adjusted for NO_2_)	−0.17 (−0.65 to 0.32)	−0.20 (−1.06 to 0.66)	−0.59 (−1.26 to 0.10)
PM_10_ (adjusted for O_3_)	−0.20 (−0.53 to 0.13)	−0.38 (−0.96 to 0.21)	−0.34 (−0.80 to 0.12)

*The conditional logistic regression model fits the pollutant(s) as unconstrained distributed lags 0–2 and adjusts for, the weekly RCGP influenza-like illness consultation rates per 100 000 England and Wales population, two natural cubic splines (df=5) for temperature (mean lag 0–1 and mean lag 2–6), public holidays and a sine/cosine annual cycle.

†Pollutant metrics: daily mean PM_2.5_, daily mean PM_10_, daily maximum 1-hour NO_2_, daily maximum 8-hour mean O_3_.

MI, myocardial infarction; NSTEMI, non-ST-elevation MI; STEMI, ST-elevation MI.

### Effect modification by season

Returning to single pollutant models, evidence of modification by season (table 2) was observed for PM_2.5_ in relation to ALL MI and for O_3_ in relation to STEMI. For O_3_ there was evidence of a negative association with STEMI in the autumn and for PM_2.5_ there was evidence of a positive association with ALL MI in the autumn. However, in both cases, there was no consistency in the direction of relative risk estimates across the four seasons.

**Table 2:** Estimates for the season-specific percentage change in risk (95% CI) per 10 µg/m^3^ increase in pollutant and tests for modification by season*: Single pollutant models†

	All MI		STEMI		NSTEMI	
Pollutant‡	Percentage change (95% CI)	p Value*	Percentage change (95% CI)	p Value*	Percentage change (95% CI)	p Value*
O_3_						
Autumn	−0.38 (−0.85 to 0.10)	0.446	−1.15 (−1.98 to −0.31)	0.040	−0.10 (−0.76 to 0.57)	0.785
Winter	0.00 (−0.37 to 0.38)		0.18 (−0.48 to 0.85)		0.04 (−0.49 to 0.56)	
Spring	0.19 (−0.29 to 0.67)		0.39 (−0.46 to 1.24)		−0.03 (−0.71 to 0.65)	
Summer	−0.07 (−0.52 to 0.38)		−0.24(−1.04 to 0.56)		−0.14 (−0.77 to 0.50)	
NO_2_						
Autumn	0.11 (−0.26 to 0.47)	0.419	−0.07 (−0.71 to 0.58)	0.060	0.13 (−0.38 to 0.64)	0.571
Winter	0.16 (−0.18 to 0.51)		0.26 (−0.34 to 0.86)		0.12 (−0.36 to 0.60)	
Spring	−0.05 (−0.39 to 0.28)		−0.55 (−1.13 to 0.03)		0.38 (−0.09 to 0.86)	
Summer	0.23 (−0.27 to 0.73)		−0.21 (−1.09 to 0.68)		0.64 (−0.07 to 1.36)	
PM_2.5_						
Autumn	0.98 (0.20 to 1.76)	0.030	0.76 (−0.62 to 2.16)	0.639	1.13 (0.05 to 2.23)	0.119
Winter	−0.70 (−1.52 to 0.13)		−0.92 (−2.35 to 0.54)		−1.13 (−2.27 to 0.02)	
Spring	−0.39 (−1.09 to 0.31)		−0.41 (−1.64 to 0.84)		−0.46 (−1.45 to 0.54)	
Summer	0.01 (−1.12 to 1.15)		−1.28 (−3.26 to 0.75)		−0.19 (−1.78 to 1.42)	
PM_10_						
Autumn	0.51 (−0.14 to 1.16)	0.155	0.53 (−0.62 to 1.70)	0.280	0.47 (−0.44 to 1.39)	0.510
Winter	−0.43 (−0.99 to 0.15)		−0.75 (−1.75 to 0.26)		−0.64 (−1.43 to 0.15)	
Spring	−0.61 (−1.20 to −0.01)		−0.26 (−1.30 to 0.80)		−0.79 (−1.62 to 0.04)	
Summer	−0.13 (−0.87 to 0.61)		−1.06 (−2.37 to 0.26)		−0.20 (−1.24 to 0.86)	

*The p values in the table relate to likelihood ratio tests for a season interaction. Seasons defined as: Autumn (September–November); winter (December–February); spring (March–May); and summer (June–August).

†The conditional logistic regression model fits the pollutant as unconstrained distributed lags 0–2 and adjusts for, the weekly RCGP influenza-like illness consultation rates per 100 000 England and Wales population, two natural cubic splines (df=5) for temperature (mean lag 0–1 and mean lag 2–6), public holidays and a sine/cosine annual cycle.

‡Pollutant metrics: daily mean PM_2.5_, daily mean PM_10_, daily maximum 1-hour NO_2_, daily maximum 8-hour mean O_3_.

MI, myocardial infarction; NSTEMI, non-ST-elevation MI; STEMI, ST-elevation MI.

### Effect modification by region

We next investigated differences in association between Government Office Regions, having adjusted for covariates at the regional level (table 3). With respect to NO_2_, PM_10_ and PM_2.5_, regression models included additional regional-level adjustment for ozone (lags 0, 1 and 2).

**Table 3:** A meta-analysis, combining Government Office Region-specific relative risk estimates* by ‘super region’ (North, Midlands, South)†

Disease	Pollutant‡ (UDLM 0–2)	Estimated percentage change in risk (95% CI) per 10 µg/m^3^ increase in pollutant‡				Test of heterogeneity between	
		North	Midlands	South	Overall	‘Super regions’ p Value	Regions p Value
All MI	O_3_	0.05 (−0.33 to 0.44)	−0.29 (−0.79 to 0.21)	−0.04 (−0.40 to 0.32)	−0.06 (−0.29 to 0.17)	0.552	0.291
	NO_2_	−0.17 (−0.50 to 0.16)	0.25 (−0.21 to 0.71)	0.21 (−0.09 to 0.51)	0.08 (−0.12 to 0.28)	0.185	0.596
	PM_2.5_	−0.51 (−1.24 to 0.22)	0.47 (−0.42 to 1.36)	0.01 (−0.61 to 0.63)	−0.06 (−0.48 to 0.36)	0.236	0.429
	PM_10_	−0.47 (−1.05 to 0.12)	0.14 (−0.56 to 0.85)	−0.18 (−0.67 to 0.31)	−0.20 (−0.53 to 0.13)	0.424	0.747
STEMI	O_3_	−0.16 (−0.85 to 0.54)	−0.36 (−1.17 to 0.47)	−0.02 (−0.66 to 0.62)	−0.15 (−0.56 to 0.26)	0.822	0.961
	NO_2_	−0.21 (−0.81 to 0.39)	−0.07 (−0.82 to 0.68)	−0.35 (−0.88 to 0.19)	−0.24 (−0.59 to 0.11)	0.838	0.994
	PM_2.5_	−1.08 (−2.39 to 0.24)	−0.07 (−1.53 to 1.41)	−0.17 (−1.27 to 0.95)	−0.42 (−1.16 to 0.31)	0.500	0.629
	PM_10_	−1.05 (−2.10 to 0.01)	−0.19 (−1.34 to 0.97)	0.01 (−0.88 to 0.89)	−0.36 (−0.95 to 0.22)	0.308	0.102
NSTEMI	O_3_	0.27 (−0.26 to 0.80)	−0.22 (−0.92 to 0.49)	−0.27 (−0.78 to 0.23)	−0.06 (−0.38 to 0.26)	0.307	0.006
	NO_2_	−0.20 (−0.67 to 0.26)	0.79 (0.13 to 1.45)	0.51 (0.09 to 0.94)	0.30 (0.01 to 0.58)	0.022	0.353
	PM_2.5_	−0.36 (−1.38 to 0.66)	0.68 (−0.58 to 1.95)	−0.42 (−1.29 to 0.45)	−0.16 (−0.75 to 0.42)	0.332	0.826
	PM_10_	−0.22 (−1.03 to 0.60)	0.38 (−0.62 to 1.39)	−0.79 (−1.48 to −0.11)	−0.36 (−0.82 to 0.11)	0.155	0.786

*Adjusted for a sine/cosine annual cycle, the weekly RCGP influenza-like illness consultation rates per 100 000 England and Wales population, region-specific natural cubic splines for temperature (mean lag 0–1 and mean lag 2–6) and public holidays. Relative risk estimates for NO_2_, PM_2.5_ and PM_10_ additionally adjusted for O_3_ (UDLM 0–2).

†**^‘^**Super regions’ were defined here by grouping three Government Office Regions to form the North (ie, North East; North West; Yorkshire and Humberside); three to form the Midlands (ie, West Midlands; East Midlands; Wales); and four to form the South (ie, South West; East; South East; London).

‡Pollutant metrics: daily mean PM_2.5_, daily mean PM_10_, daily maximum 1-hour NO_2_, daily maximum 8-hour mean O_3_.

MI, myocardial infarction; NSTEMI, non-ST-elevation MI; STEMI, ST-elevation MI; UDLM, unconstrained distributed lag model.

For ALL MI and for STEMI there was no evidence of heterogeneity in association between regions or between ‘super regions’ (North, Midlands, South) whether in relation to O_3_, NO_2_, PM_2.5_ or PM_10_. In contrast for NO_2_ and NSTEMI, subtotal relative risks for the Midlands and the South were significant and >1, suggestive of increases of 0.79% (0.13% to 1.45%) and 0.51% (0.09% to 0.94%) per 10 µg/m^3^, respectively, whereas in the North the subtotal relative risk was <1 and non-significant (test for heterogeneity between ‘super regions’, p=0.022). For O_3_ and NSTEMI, associations differed significantly between regions (p=0.006) but not between ‘super regions’.

When we compared the overall estimates (ie, over all regions) in table 3 with their corresponding values in table 1, it appeared that adjustment for region had little effect.

### PM_2.5_ components

Table 4 displays the results from investigating the associations between our three outcomes and two selected PM_2.5_ components. The two components tabulated are EC (emitted from combustion) and sulfate (SO_4_^2−^, formed within the atmosphere from SO_2_). The conditional logistic regression models adjust for temperature at lags 0–1 and 2–6 days, public holidays, influenza and residual seasonality. Neither EC nor SO_4_^2−^ was associated with ALL MI, STEMI or NSTEMI either before or after additional adjustment for O_3_ at lags 0, 1 and 2.

**Table 4:** Estimates for the percentage change in risk (95% CI) per one IQR* increase in pollutant: single and two pollutant models†

PM_2.5_ components	All MI Percentage change (95% CI)	STEMI Percentage change (95% CI)	NSTEMI Percentage change (95% CI)
Single pollutant regression model			
EC	0.10 (−0.18 to 0.37)	−0.15 (−0.64 to 0.33)	0.13 (−0.26 to 0.52)
SO_4_^2−^	−0.02 (−0.30 to 0.27)	−0.29 (−0.79 to 0.21)	−0.08 (−0.48 to 0.32)
Two pollutant regression model			
EC (adjusted for O_3_)	0.06 (−0.24 to 0.35)	−0.28 (−0.79 to 0.24)	0.11 (−0.30 to 0.52)
SO_4_^2−^ (adjusted for O_3_)	−0.02 (−0.31 to 0.26)	−0.31 (−0.82 to 0.19)	−0.09 (−0.49 to 0.31)

*IQR for daily mean EC=0.213 µg/m^3^ and IQR for daily mean SO_4_^2−^=1.710 µg/m^3^.

†The conditional logistic regression model fits the pollutant(s) as unconstrained distributed lags 0–2 and adjusts for, the weekly RCGP influenza-like illness consultation rates per 100 000 England and Wales population, two natural cubic splines (df=5) for temperature (mean lag 0–1and mean lag 2–6), public holidays and a sine/cosine annual cycle.

EC, elemental carbon; MI, myocardial infarction; NSTEMI, non-ST-elevation MI; STEMI, ST-elevation MI.

### Sensitivity analysis

**Figure 1:**
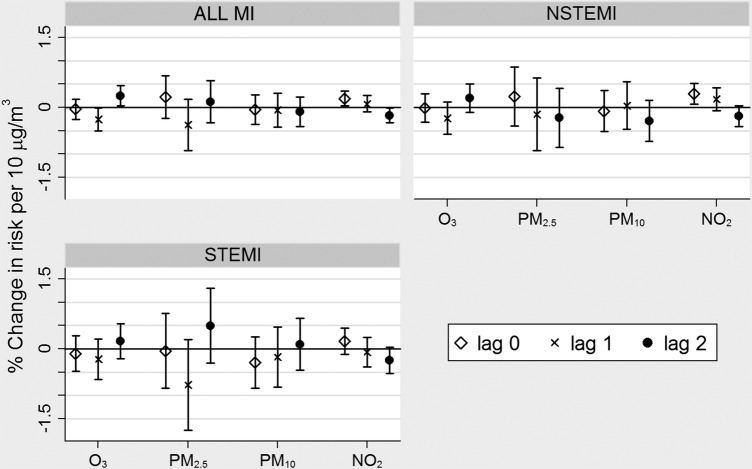
Associations between atmospheric chemistry transport model pollution concentrations at lags 0, 1 and 2 and myocardial infarction (% change in risk per 10 µg/m^3^ increase in pollutant). Pollutant metrics: daily mean PM_2.5_, daily mean PM_10_, daily maximum 1-hour NO_2_, daily maximum 8-hour mean O_3_.

As part of our sensitivity analyses, we returned to our single pollutant regressions (table 1) and plotted the individual components of each UDLM as the percentage change in risk per 10 µg/m^3^. From figure 1, it appeared that the association between NO_2_ and NSTEMI was primarily due to concentrations on the day (lag 0) rather than on the 1 day (lag 1) or 2 days prior (lag 2).

Next we looked for evidence of non-linearity in the associations between simple pollution averages (across lags 0, 1 and 2) and the log relative risks of ALL MI, STEMI and NSTEMI. Having adjusted for covariates, we found no evidence of non-linearity based on Wald tests with 1 df (all p>0.19).

Finally, to investigate the sensitivity of our findings to the level of control for seasonality, we re-ran our single pollutant regression models without the sine and cosine terms and observed little change in risk estimates and CIs (see online supplementary table S2).

## Discussion

### Main findings

We investigated associations between gaseous and particulate air pollution and three outcomes, and found no evidence of associations except for one positive association between modelled NO_2_ and NSTEMI, suggestive of a 0.27% increase in NSTEMI per 10 µg/m^3^ increase in daily maximum 1-hour NO_2_ (UDLM 0–2). This association increased in magnitude and statistical significance following adjustment for PM_2.5_. While not significant in any one season, the relative risk for NO_2_ and NSTEMI in our single pollutant model was consistently >1 in the autumn, winter, spring and summer months (table 2). However, this consistency was not replicated in our regional analysis, which suggested that any positive association between NO_2_ and NSTEMI was confined to the Midlands and the South (table 3). The lack of association with EC and SO_4_^2−^ is noteworthy given their novelty and what they represent.

### Comparison with other studies

A recent systematic review of air pollution and MI^10^ identified 34 time-series/case-crossover studies published between 1997 and 2011 for inclusion in meta-analyses. The studies were mainly from the USA and Europe (including two from the UK) and varied in their source of outcome data (eg, hospital admissions, death registries, MI registries). In common with our results, the review found no evidence of an association between MI and O_3_ (lag 0). However, it reported statistically significant increases in the risk of MI of 1.1%, 0.6% and 2.5% per 10 µg/m^3^ increases in NO_2_ (lag 1), PM_10_ (lag 0) and PM_2.5_ (lag 1), respectively. These represent very different relative risks to those observed (UDLM 0–2) in our study (0.09%, −0.20%, −0.04%, respectively). In particular, the increase in risk for PM_2.5_ is not apparent in our study. One possible explanation for the difference between our finding and those from the review (besides obvious differences of time-period, geography and outcome measure) is our use of modelled rather than monitored pollutant data. We, therefore, compared our findings with those of a previously published analysis of the MINAP database (2003–2009) by Milojevic *et al*,^11^ which investigated associations between MI and nearest monitor pollution concentrations (up to 50 km distant from residential address). In common with our own results, they found no evidence of positive associations with O_3_, PM_2.5_ or PM_10_ but a significant positive association of daily mean NO_2_ (lags 0–4) with NSTEMI, although their estimated increase in risk was more marked (0.68% per 10 µg/m^3^ increase in NO_2_ (95% CI 0.07% to 1.3%)). One possible explanation for this difference is the different uncertainties in the monitor-based pollution data used by Milojevic *et al* (measurements made up to 50 km from residential address) and the model-based exposure assessment used here (see online supplementary table S1).

### Strengths and limitations

One of the strengths of using MINAP data is that it provides us with a large number of events and hence good statistical power for detecting associations with ALL MI but also with the MI subtypes of STEMI and NSTEMI. However, given the consideration of three outcomes and six pollutants in our analyses, the possibility of obtaining a spurious significant association cannot be discounted.

We acknowledge the potential for modelled pollution concentrations based on any ACTM to introduce more classical measurement error into a time series analysis than monitor data and hence lead to greater attenuation in relative risk estimates. However, based on our comparison of model and monitor data in online supplementary table S1 and our previous statistical simulation study,^24^ we expect this to be less of an issue for O_3_ and for urban PM_2.5_. Measurement error (classical and Berkson) may also lead to a reduction in statistical power, although, for ACTMs, the ability to provide more complete pollutant time series may partly compensate by limiting any reduction in sample size due to missing data.

One advantage of using modelled pollution data is that we can obtain geographically complete data, as measurements of PM_2.5_ and other species are limited (in space and time) over the UK. In addition, models can provide particulate matter component data, which, in England and Wales, are sparsely monitored, or restricted to intensive field campaigns, and hence for which little historical data are available. While, in this study, we found no evidence of associations with the EC or sulfate components of PM_2.5_, there may be associations with other particle component matter not yet investigated.

### Critical exposure period

If there is a detrimental effect of some pollutants, it is not clear whether these effects are immediate (in the few hours prior to the event), delayed or cumulative. A small study of MI and particulate air pollution in New York reported a positive association of STEMI with PM_2.5_ exposure in the 1 hour prior to event, with ORs for the 3, 12 and 24 hours prior to event, >1 but non-significant.^12^ Similarly, a large, though exploratory, study of air pollution and acute MI in Alberta, Canada, reported positive associations of NSTEMI in the 65 and over age group with average NO_2_ exposures in the 6, 12 and 24 hours prior to event (suggesting elevations in risk of 3.7%, 3.8% and 3.7% per 10 µg/m^3^, respectively).^13^

In terms of MINAP, a previous study of the database (2003–2006) by Bhaskaran *et al*,^28^ found evidence of positive associations between MI and NO_2_ and PM_10_ exposure in the 1–6 hours prior to event (suggesting elevations in risk of 1.1% and 1.2% per 10 µg/m^3^, respectively) but no evidence of associations with exposures in the 7–12, 13–18, 19–24 or 25–72 hours prior. While, in contrast, the Milojevic *et al*,^11^ study found stronger associations of all MI and NSTEMI with NO_2_ at lags 0–4 days rather than NO_2_ at lags 0–1 days, our sensitivity analyses with modelled pollutant data (figure 1) suggested stronger associations with the more recent exposure (ie, lag 0 days) than lags 1 or 2 days.

### Mechanisms

Much has been written and hypothesised as to the various possible mechanisms by which an increase in the level of exposure to certain pollutants may increase the risk of an acute coronary event, including by increasing blood pressure, blood viscosity, promoting an inflammatory response, interfering with heart rhythm or promoting vasoconstriction.^3^ ^4^ Of interest here is how these mechanisms may differentially impact on STEMI and NSTEMI. The total occlusion of a coronary artery characteristic of STEMI is more likely to result from plaque rupture and thrombus formation than the partial occlusion characteristic of NSTEMI.^12^ ^29^ Gardner *et al*,^12^ therefore, suggested that whether exposure to a given pollutant (in their case PM_2.5_) was more important in the aetiology of STEMI than NSTEMI may depend on whether or how that pollutant influences the processes leading to thrombus formation and/or thrombus dissolution.

## Conclusion

In common with a previous study of the MINAP database,^11^ but using a different data set with notably wider geographical coverage, modelled temperature and pollution data modelled by an atmospheric chemistry transport model, we found some evidence of a positive association between exposure to NO_2_ and the risk of NSTEMI. The appropriate exposure period or lag to consider is still unclear and more immediate effects of other pollutants in the hours rather than the day of and or days prior to event may have been missed. Given our study detected only one positive association, it is possible that this finding is simply an artefact. Confirmation of any such association in databases other than MINAP is therefore required.

## References

[1] World Health Organisation. *Review of evidence on health aspects of air pollution—REVIHAAP project: Technical report*. WHO Regional Office for Europe 2013. http://euro.who.int/en/health-topics (accessed 9 Mar 2015).

[2] Committee on the Medical Effects of Air Pollutants (COMEAP). Cardiovascular disease and air pollution. London: Department of Health, 2006 http://www.comeap.org.uk/documents (accessed 9 Mar 2015).

[3] Brook RD, Rajagopalan S, Pope CA III, et al Particulate matter air pollution and cardiovascular disease: an update to the scientific statement from the American Heart Association. Circulation 2010;121:2331–78. 10.1161/CIR.0b013e3181dbece1 20458016

[4] Bhaskaran K, Wilkinson P, Smeeth L Cardiovascular consequences of air pollution: what are the mechanisms? Heart 2011;97:519–20. 10.1136/hrt.2010.212183 21148574

[5] Nuvolone D, Balzi D, Pepe P Ozone short-term exposure and acute coronary events: a multicities study in Tuscany (Italy). Environ Res 2013;126:17–23. 10.1016/j.envres.2013.08.002 24011457

[6] Goggins WB, Chan EYY, Yang C-Y Weather, pollution, and acute myocardial infarction in Hong Kong and Taiwan. Int J Cardiol 2013;168:243–9. 10.1016/j.ijcard.2012.09.087 23041014

[7] Cadum E, Berti G, Biggeri A [The results of EpiAir and the national and international literature]. Epidemiol Prev 2009;33(Suppl 1):113–19, 123–43. 20418591

[8] Maitre A, Bonneterre V, Huillard L Impact of urban atmospheric pollution on coronary disease. Eur Heart J 2006;27:2275–84. 10.1093/eurheartj/ehl162 16893917

[9] Bhaskaran K, Hajat S, Hains A Effects of air pollution on the incidence of myocardial infarction. Heart 2009;95:1746–59. 10.1136/hrt.2009.175018 19635723

[10] Mustafić H, Jabre P, Caussin C Main air pollutants and myocardial infarction: a systematic review and meta-analysis. JAMA 2012;307:713–21. 10.1001/jama.2012.126 22337682

[11] Milojevic A, Wilkinson P, Armstrong B Short-term effects of air pollution on a range of cardiovascular events in England and Wales: case-crossover analysis of the MINAP database, hospital admissions and mortality. Heart 2014;100:1093–8. 10.1136/heartjnl-2013-304963 24952943 PMC4078678

[12] Gardner B, Ling F, Hopke PK Ambient fine particulate air pollution triggers ST-elevation myocardial infarction, but not non-ST elevation myocardial infarction: a case-crossover study. Part Fibre Toxicol 2014;11:1 10.1186/1743-8977-11-1 24382024 PMC3891992

[13] Wang X, Kindzierski W, Kaul P Air pollution and acute myocardial infarction hospital admission in Alberta, Canada: a three-step procedure case-crossover study. PLoS ONE 2015;10:e0132769 10.1371/journal.pone.0132769 26167938 PMC4500548

[14] Herrett E, Smeeth L, Walker L The Myocardial Ischaemia National Audit Project (MINAP). Heart 2010;96:1264–7. 10.1136/hrt.2009.192328 20659944 PMC3505836

[15] Vieno M, Dore AJ, Stevenson DS Modelling surface ozone during the 2003 heat-wave in the UK. Atmos Chem Phys 2010;10:7963–78. 10.5194/acp-10-7963-2010

[16] Vieno M, Heal MR, Hallsworth S The role of long-range transport and domestic emissions in determining atmospheric secondary inorganic particle concentrations across the UK. Atmos Chem Phys 2014;14:8435–47. 10.5194/acp-14-8435-2014

[17] Simpson D, Benedictow A, Berge H The EMEP MSC-W chemical transport model—technical description. Atmos Chem Phys 2012;12:7825–65. 10.5194/acp-12-7825-2012

[18] NICOR. Datasets and user guides. https://www.ucl.ac.uk/nicor/audits/minap/datasets (accessed 16 May 2016).

[19] Weather Research and Forecasting (WRF) model version 3.1.1. http://www.wrf-model.org (accessed 26 Jan 2016).

[20] UK National Atmospheric Emissions Inventory (NAEI). © Crown 2015 copyright Defra & DECC via naei.defra.gov.uk, licenced under the Open Government Licence (OGL) http://www.nationalarchives.gov.uk/doc/open-government-licence/version/2/ (accessed 24 Aug 2015).

[21] Centre for Emissions Inventories and Projections (CEIP). http://www.ceip.at (accessed 26 Jan 2016).

[22] Automatic Urban and Rural Monitoring Network (AURN) Data Archive. © Crown 2015 copyright Defra via uk-air.defra.gov.uk, licenced under the Open Government Licence (OGL) http://www.nationalarchives.gov.uk/doc/open-government-licence/version/2/ (accessed 9 Jun 2015). 10.1007/s12630-012-9853-y

[23] Carslaw D Defra regional and transboundary model evaluation analysis—phase 1, a report for Defra and the Devolved Administrations 2011. http://uk-air.defra.gov.uk/reports/cat20/1105091514_RegionalFinal.pdf (accessed 3 Feb 2015).

[24] Butland BK, Armstrong B, Atkinson RW Measurement error in time-series analysis: a simulation study comparing modelled and monitored data. BMC Med Res Methodol 2013;13:136 10.1186/1471-2288-13-136 24219031 PMC3871053

[25] Bhaskaran K, Hajat S, Hains A Short term effects of temperature on risk of myocardial infarction in England and Wales: time series regression analysis of the Myocardial Ischaemia National Audit Project (MINAP) registry. BMJ 2010;341:c3823 10.1136/bmj.c3823 20699305 PMC2919679

[26] Janes H, Sheppard L, Lumley T Case-crossover analyses of air pollution exposure data: referent selection strategies and their implications for bias. Epidemiology 2005;16:717–26. 10.1097/01.ede.0000181315.18836.9d 16222160

[27] Office for National Statistics and the Royal College of General Practitioners Research and Surveillance Centre. Weekly deaths from all causes and RCGP influenza-like illness (ILI) consultation rates per 100,000 population, England and Wales, 1999–2013: in Excess Winter Mortality in England and Wales, 2012/13 (Provisional) and 2011/12 (Final). http://www.ons.gov.uk/ons/rel/subnational-health2/excess-winter-mortality-in-england-and-wales/2012-13--provisional--and-2011-12--final-/index.html (accessed 29 Oct 2014) Licenced under the Open Government Licence v3.0 http://www.nationalarchives.gov.uk/doc/open-government-licence

[28] Bhaskaran K, Hajat S, Armstrong B The effects of hourly differences in air pollution on the risk of myocardial infarction: case crossover analysis of the MINAP database. BMJ 2011;343:d5531 10.1136/bmj.d5531 21933824 PMC3176903

[29] Hong YJ, Jeong MH, Choi YH Differences in intravascular ultrasound findings in culprit lesions in infarct-related arteries between ST segment elevation myocardial infarction and non-ST segment elevation myocardial infarction. J Cardiol 2010;56:15–22. 10.1016/j.jjcc.2010.01.010 20350520

