# Supplementary material for: Myocardial infarction, ST-elevation and non-ST-elevation myocardial infarction and modelled daily pollution concentrations: a case-crossover analysis of MINAP data
Source: Open Heart. 2016 Sep 1;3(2):e000429. doi: 10.1136/openhrt-2016-000429 (PMC5013456; doi:10.1136/openhrt-2016-000429)
Supplement: Supplementary data [file openhrt-2016-000429supp.pdf]

Supplementary Table 1 Comparing monitored pollutant concentrations 2001-2010 at AURN monitoring sites* with their corresponding modelled equivalents

Pollutant†	Site Type	No. of monitoring sites	Average within-site standard deviation		Average within-site covariance $Cov(mon, mod)$	Average within-site correlation $Corr(mon, mod)$	Estimated attenuation in logistic regression coefficient due to CTM measurement error‡
			Monitor $sd(mon)$	Model $sd(mod)$			
**O_3**	Rural Background	24	20.334	19.731	296.075	0.725	24%
	Urban Background	63	23.189	22.071	392.185	0.758	19%
**NO_2**	Rural Background	16	14.110	17.608	170.796	0.651	45%
	Urban Background	75	23.425	23.343	301.655	0.542	45%
**PM_{10}**	Rural Background	5	7.764	12.417	46.531	0.469	70%†
	Urban Background	57	10.587	12.762	67.506	0.501	59%
**$PM_{2.5}$**	Rural Background	3	5.158	8.437	32.313	0.733	55%†
	Urban Background	39	8.618	6.756	39.696	0.686	13%

*(Source: Automatic Urban and Rural Monitoring Network (AURN) Data Archive. © Crown 2015 copyright Defra via uk-air.defra.gov.uk, licenced under the Open Government Licence (OGL))

†Pollutant metrics: daily mean $PM_{2.5}$, daily mean PM_{10}, daily maximum1-hour NO_2, daily maximum 8-hour mean O_3. The monitoring of $PM_{2.5}$ only began part-way through the comparison period of 2001-2010. Between 2001 and 2010 there were instrument changes in the monitoring of PM_{10}.

‡Estimated attenuation = $\left[1 - \left\{\frac{cov(mon,mod)}{var(mod)}\right\}\right] \times 100\%$ [24] (Measurement error is assumed to be additive).

Supplementary Figure 1

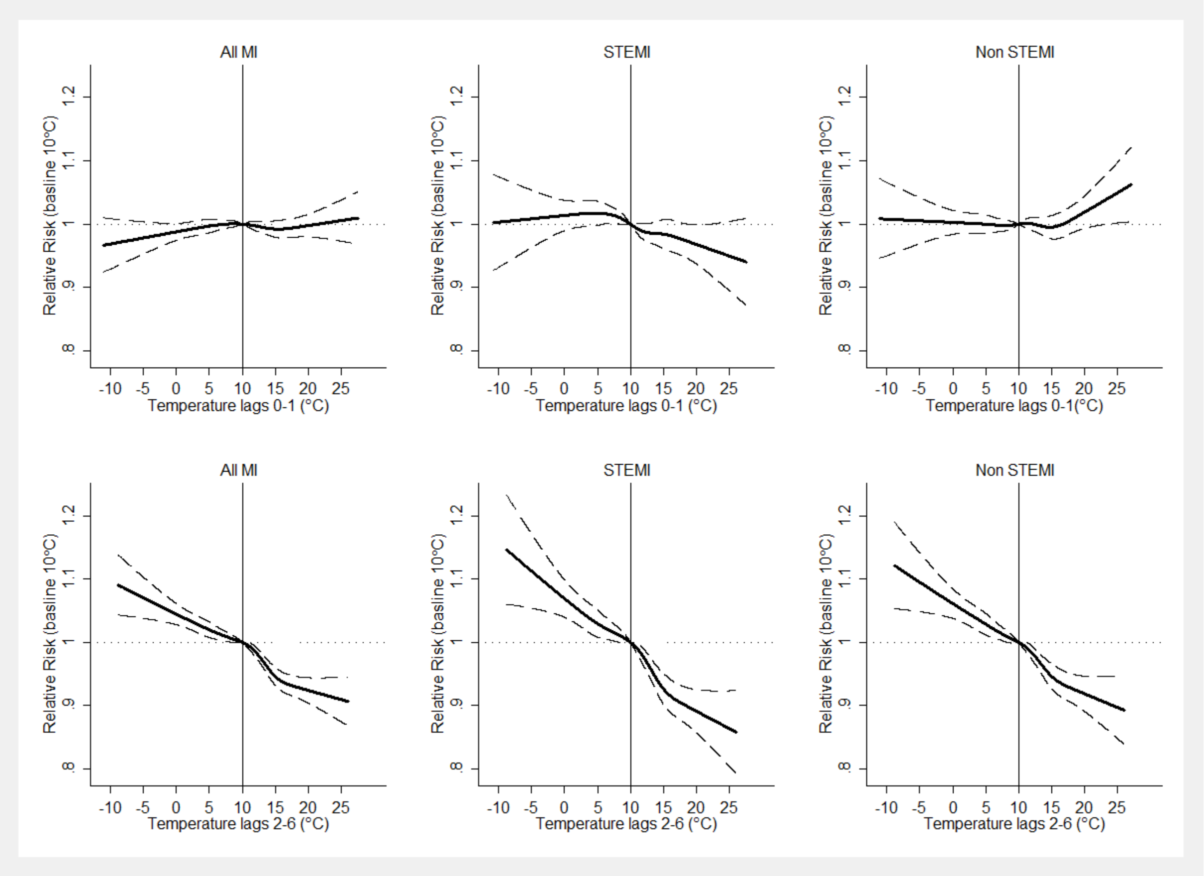

Investigating the associations of myocardial infarction (ALL MI), ST-elevation myocardial infarction (STEMI) and non-ST-elevation myocardial infarction (NSTEMI) with mean daily temperature averaged over lags 0-1 days and over lags 2-6 days.

Supplementary Table 2: Estimates of the percentage change in risk [95% CI] per 10 μg/m^3 increase in pollutant: Single pollutant models* (no adjustment for sine cosine annual cycle)

**Pollutant**[†]	**All MI**<br>**% change [95% CI]**	**STEMI**<br>**% change [95% CI]**	**NSTEMI**<br>**% change [95% CI]**
**Single pollutant regression model**			
O_3	-0.07 [-0.30, 0.16]	-0.21 [-0.61, 0.19]	-0.03 [-0.35, 0.29]
NO_2	0.10 [-0.09, 0.29]	-0.13 [-0.46, 0.20]	0.27 [0.01,0.54]
$PM_{2.5}$	-0.03 [-0.44, 0.38]	-0.35 [-1.07, 0.38]	-0.14 [-0.71, 0.44]
PM_{10}	-0.24 [-0.57, 0.08]	-0.46 [-1.03, 0.12]	-0.33 [-0.78, 0.13]

*The conditional logistic regression model fits the pollutant(s) as unconstrained distributed lags 0-2 and adjusts for, the weekly RCGP influenza-like illness consultation rates per 100,000 England and Wales population, two natural cubic splines (df=5) for temperature (mean lag 0-1 and mean lag 2-6), public holidays.

[†]Pollutant metrics: daily mean $PM_{2.5}$, daily mean PM_{10}, daily maximum1-hour NO_2, daily maximum 8-hour mean O_3.
